# Willingness and Behaviors of Farmers’ Green Disposal of Pesticide Packaging Waste in Henan, China: A Perceived Value Formation Mechanism Perspective

**DOI:** 10.3390/ijerph17113753

**Published:** 2020-05-26

**Authors:** Mingyue Li, Jingjing Wang, Kai Chen, Lianbei Wu

**Affiliations:** School of Economics and Management, Beijing Forestry University, Beijing 100083, China; limingyue_2019@bjfu.edu.cn (M.L.); wangjingj97@bjfu.edu.cn (J.W.); wulianbei@bjfu.edu.cn (L.W.)

**Keywords:** perceived benefits, perceived risks, green disposal willingness, green disposal behaviors, structural equation model (SEM)

## Abstract

Environmental pollution as a result of the improper disposal of pesticide packaging wastes (PPWs) has posed serious harm to groundwater, soil and public health. However, few studies focused on PPWs green disposal willingness and behaviors of farmers from the perspective of perceived value. Based on the first-hand data, collected from 635 farmers of grain-producing counties in Henan province of China, through the questionnaire survey method, this paper adopted a structural equation model (SEM) to empirically explore the formation mechanism of perceived value on PPWs green disposal, and green disposal willingness and behaviors were further in-depth investigated. The results showed that the action of farmers’ green disposal of PPWs followed the causal relationship, whereby perceived value→behavioral willingness→behavioral performance, and farmers’ perceived value came from the comprehensive tradeoff and comparison between perceived benefits and perceived risks. Meanwhile, the perceived benefits and perceived risks could have significant effects on green disposal willingness and behaviors directly and indirectly, among which perceived benefits (0.478) had the greatest positive total effects on the willingness, and perceived risks (−0.362) had the greatest negative total effects on the behaviors. Interestingly, there existed inconsistence between farmers’ green disposal willingness and behaviors. When faced with the choice of PPWs green disposal, the farmers were generally risk averse, which resulted in them being more inclined to take conservative behaviors driven by the profit maximization, and even showed the “powerless” state with willingness but no actual action.

## 1. Introduction

Pesticides play a positive role in ensuring the increase of agricultural output and farmers’ income, as well as the effective supply of agricultural products, and have an important inputs in agriculture [1,2]. China’s total agricultural output has increased, and pesticides have played an irreplaceable role in maintaining its high yield [3]. Nonetheless, farmers tend to overuse pesticides to better control the crop diseases and pests [4,5], which were accompanied by the improper disposal of amounts of pesticide packaging waste (PPWs) resulting in environmental pollution that had become one of the outstanding problems of agricultural non-point source pollution in China [6,7]. PPWs mainly refer to the packaging materials directly in contact with pesticides discarded after use in the agriculture production, including bottles, cans, barrels and bags made of plastic, glass, metal, paper and other materials [8]. In order to control the pollution caused by PPWs, the State Council of China promulgated the newly revised Regulations on Pesticide Management (RPM) in 2017 to clarify the responsibility subjects and important obligations of recycling PPWs for the first time. Subsequently, pilot action of PPWs recycling was carried out in Zhejiang, Shandong, Henan and other provinces in succession focusing on ecological environment quality and agricultural product supply safety. 

However, PPWs recycling mechanism in China is still in the exploration stage, and farmers generally have a low awareness of recycling policies and measures, which weakens the implementation effect of relevant policies to some extent [9]. Meanwhile, the deterioration of rural environment and agricultural non-point source pollution were aggravated due to the lack of waste disposal facilities, relatively weak environmental awareness of farmers and non-standard disposal methods and inherent habits in the vast rural areas [10,11]. It was reported that China needs 10.4 billion pesticide packages per year, and 3.2 billion of them are discarded randomly with a total weight of over 100,000 tons. Nonetheless, residual pesticide in these packages caused irreversible harm to underground water, soil structures, environmental organisms and human health [12,13,14,15]. 

The impact of PPWs on ecological environment was an important research hotspot, and it was generally assumed that the green disposal of PPWs is not only related to the realization of agricultural ecological value, but also affects the health of rural resident [16,17]. The “green disposal” refers to a behavioral pattern of handling the pesticide packaging waste by adhering to the development concept of “green”. In addition, ‘green’ in “green disposal” is quite similar to the concept of “sustainability” in that it means reducing environmental pollution, saving resources and promoting health. Increasingly, researchers have substantively addressed PPWs disposal behaviors, and basically concluded that farmers often did not send PPWs to government recycling centers or pesticide supply and marketing centers [18]. Instead, PPWs were usually thrown into the fields and ditches [19], or burned in the open, buried in the farm [20] or placed the general rubbish [21], this kind of inappropriate disposal behaviors commonly happened in the developing countries [14,22]. 

To explore the underlying causes of above behaviors, researchers have analyzed the key factors affecting farmers’ behaviors of PPWs disposal. Researches showed that farmers’ behavior decision-making of PPWs disposal was the results of multiple factors including individual characteristics (age, education level, marital status) [23,24], family endowment (quantity of labor force, arable land area, farming experience) [22], social responsibility (satisfaction degree of farmers on agricultural activities) [25], geographical location (distance between village and the pesticides service center or the city) [26], and cognition characteristics (knowledge of pesticides, awareness of risks) [27]. These have all had a significant impact on PPWs disposal behaviors. In addition, Huang et al. [28] sorted out the mature recycling models of PPWs, and believed that the government-led superfund system for pollution control in the United States and the market-oriented green point waste recycling management system in Germany were representative for now. Nevertheless, Li and Huang [8] explored the recycling and utilization mechanism of PPWs from the perspective of reverse logistics, as well as the positive impacts of the reverse logistics mechanisms on the ecological and social benefits. Geographic information system (GIS) technology was used by the researcher to assess the generation of solid waste [29].

There are still some aspects of the above findings that need further investigation. First, the existing research mainly discussed the factors influencing farmers’ PPWs disposal behaviors from the angle of demographic characteristics [22,24], environmental cognitive characteristics [26,27], social system characteristics [9,28], etc. However, few of them introduced psychological factors, such as perceived value to investigate the psychological decision-making mechanism of farmers’ PPWs disposal. According to relevant studies, attitude is the primary factor influencing their behavioral willingness [30], while perceived value is the most direct reason for the formation of behavioral attitude [31]. Farmers’ green disposal behaviors of PPWs largely depend on their perceived value. Therefore, the research explored farmers’ disposal willingness of PPWs from the perspective of perceived value, which clarified the psychological mechanism and behavioral logic of the farmers’ green disposal of PPWs, and this significant in standardizing farmers on the PPWs green disposal. Second, previous studies mostly used discrete selection models such as Logit [26] or Probit [11] to analyze the direct influence of each independent explanatory variable on the farmers’ disposal behaviors of PPWs. Nevertheless, few studies have adopted a structure equation model (SEM) to deeply explore the action path and internal mechanism of each influencing factor. The SEM model [30,32,33] was used to study the social psychological mechanism behind farmers’ willingness and behaviors to PPWs green disposal, which not only identified the factors hindering farmers’ willingness, but also clarified the mechanism involved in promoting farmers’ behaviors. In addition, there are few studies targeting the specific field of farmers’ willingness and behaviors to PPWs green disposal.

In view of the above analysis, the main objective of this study was to draw lessons from theory of perceived value, introduced the psychological variables, utilized the survey data of 635 farmers from the major grain-producing counties of Henan province of China and adopted the SEM model to investigate farmers’ willingness and behaviors of PPWs green disposal. The specific purposes of this study are to; (i) validate the formation mechanism of farmers’ perceived value in the PPWs green disposal; (ii) further investigate the effects of farmers’ perceived value on PPWs green disposal behaviors. These would contribute to provide references for the government to identify the farmers’ PPWs green disposal behavior characteristics and formulate relevant policies to control the agricultural pollution.

## 2. Theoretical Background and Research Hypothesis

Zeithaml (1988) formally put forward the theory of perceived value (TPV), arguing that customer perceived value is a subjective and comprehensive evaluation of a product, service or behavior, through the tradeoff and comparison between the benefits and costs, based on the individual cognition from the perspective of individual experience [34]. As for the formation mechanism of perceived value, the “hierarchical model” is believed that perceived value comes from the individual’s processing of perceived information. It is based on the comparison between the expectation before behaviors and the results after behaviors in terms of the individual’s cognitive logics of consumer products, services and other factors [35,36]. As for the influence of perceived value on the behavioral willingness and decision-making, the “tradeoff model” believed that perceived value is an individual’s subjective evaluation of the tradeoff between gains (benefits) and losses (risks) [37]. When the perceived gains (such as product gains and emotional satisfaction) are greater than the perceived losses (such as currency losses and opportunity costs), the higher the individual perceived value level, the more obvious their behavioral tendency [38]. 

**Figure 1:**
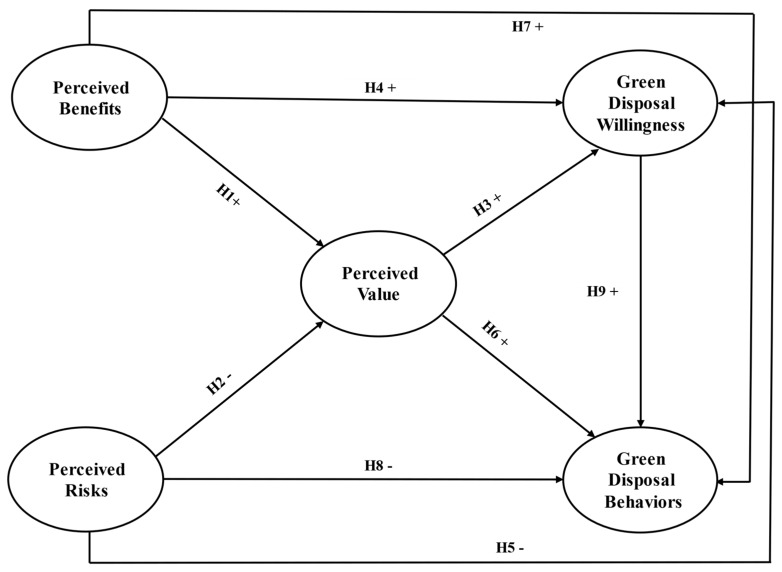
Theoretical Model.

Based on the above analysis, the theory of perceived value clearly illustrates the path pattern and logic mechanism of individual behavior decision-making, namely cognitive level→cognitive tradeoff→perceived value→behavior willingness→behavior performance, which provides good theoretical support for the investigation of farmers’ PPWs green disposal behaviors from the perspective of TPV. Therefore, this study constructed a theoretical model by combining the research results of TPV and farmer behaviors as shown in Figure 1. In the model, farmers could make subjective cognitive evaluation on the value of PPWs green disposal, based on their own situation, after weighing the benefits and risks of PPWs green disposal. The model includes two antecedent variables of perceived value, that is, perceived benefits and perceived risks. It also includes two outcome variables of the perceived value, namely the green disposal willingness and the green disposal behaviors, indicating that the perceived value could affect the farmers’ green disposal willingness and behaviors of PPWs. 

### 2.1. Factors Influencing Farmers’ Perceived Value

The farmers’ perceived value of PPWs green disposal is a subjective and comprehensive evaluation, obtained by comparing the perceived benefits and risks in the decision-making process. Based on the “tradeoff model” of TPV, farmers’ perceived benefits and risks have an impact on farmers’ perceived value [31,37,39]. Farmers’ perceived benefits refer to the gains that are perceived subjectively in PPWs green disposal, such as economic benefits, environmental benefits, health benefits and resource conservation [40,41,42]. Farmers’ perceived risks refer to the losses that are perceived subjectively in PPWs green disposal, such as currency expenditure, opportunity cost, time risk and labor cost [31,43]. For PPWs green disposal, the primary factor to consider whether farmers will participate in green disposal or not is the gains they get before implementing green disposal behaviors. The farmers’ perceived value is higher if they expect to gain more and pay less, and vice versa. Therefore, the following hypotheses are proposed:

**Hypothesis 1** **(H1).** *Perceived benefits have a significantly positive impact on farmers’ perceived value.*

**Hypothesis 2** **(H2).** *Perceived risks have a significantly negative impact on farmers’ perceived value.*

### 2.2. Influence of Perceived Value on Farmers’ Willingness to Green Disposal

According to theory of planned behavior, individual behaviors are thoughtful and planned, and various factors indirectly affect individual behavioral decisions through willingness [44,45]. This provides a theoretical basis for understanding how farmers change their behavioral decisions. In the decision-making process, farmers’ green disposal willingness refers to their psychological intention on PPWs green disposal. In general, farmers have a higher behavioral willingness when they expect the benefits to be greater than the costs; farmers will have a lower willingness when they expect the benefits to be less than the costs [46,47]. In addition, some researchers pointed out that the higher the farmers’ perceived value, the higher their participation willingness [48,49]. That is, farmers’ perceived value has positive promotion effects on their willingness to PPWs green disposal. Therefore, the following hypotheses are proposed:

**Hypothesis 3** **(H3).** *Perceived value has a significantly positive impact on farmers’ green disposal willingness*.

**Hypothesis 4** **(H4).** *Perceived benefits have a significantly positive impact on farmers’ green disposal willingness.*

**Hypothesis 5** **(H5).** *Perceived risks have a significantly negative impact on farmers’ green disposal willingness.*

### 2.3. Influence of Perceived Value on Farmers’ Green Disposal Behaviors

Previous studies have shown that individual behavioral decisions involves a comprehensive judgment after weighing and comparing the result utility of benefits and costs [50,51,52]. For PPWs green disposal, perceived value level is the main factor to decide whether to implement green disposal behaviors. When farmers expected more gains in the PPWs green disposal behaviors, namely the PPWs green disposal behaviors can obtain better utility, perceived value level will be higher, and they are more inclined to adopt the green disposal behaviors; On the contrary, when farmers expected the gains to be less than the losses in the PPWs green disposal behaviors, namely when the results of PPWs green disposal cannot achieve utility and cause losses, the farmers’ perceived value level will be lower, and they are more inclined to give up the green disposal behaviors. Other studies about farmers’ emotions showed that perceived value had a significant impact on their regret mood tendency [53]. Research results in the field of green product marketing indicated that perceived value could positively affect individuals’ purchase behaviors [33,54]. Therefore, the following hypotheses are proposed:

**Hypothesis 6** **(H6).** *Perceived value has a significantly positive impact on farmers’ green disposal behaviors.*

**Hypothesis 7** **(H7).** *Perceived benefits have a significantly positive impact on farmers’ green disposal behaviors.*

**Hypothesis 8** **(H8).** *Perceived risks have a significantly negative impact on farmers’ green disposal behaviors.*

### 2.4. Influence of Green Disposal Willingness on Farmers’ Green Disposal Behaviors

PPWs green disposal behaviors refer to farmers’ ecological and environmental protection behaviors in the agricultural production process concerning the disposing PPWs in a “green” way, such as “collected and sold them to recycling buyers”, “collected and threw them to garbage centralized treatment”, “collected and sent them to the agricultural capital supply and marketing center”, etc. Some studies pointed out that behavioral willingness is the most direct factor of behavioral achievement [55]. The stronger an individual’s willingness to perform a certain behavior, the more likely it is to promote the behavior implementation [44,56]. Similarly, farmers’ green disposal willingness affects their green disposal behaviors in the decision-making process of the PPWs green disposal, namely the higher the behavioral willingness, the more active the implementation. Therefore, the following hypothesis is proposed:

**Hypothesis 9** **(H9).** *Green disposal willingness has a significantly positive impact on farmers’ green disposal behaviors.*

## 3. Materials and Methods

### 3.1. Study Area and Sample Collection

**Figure 2:**
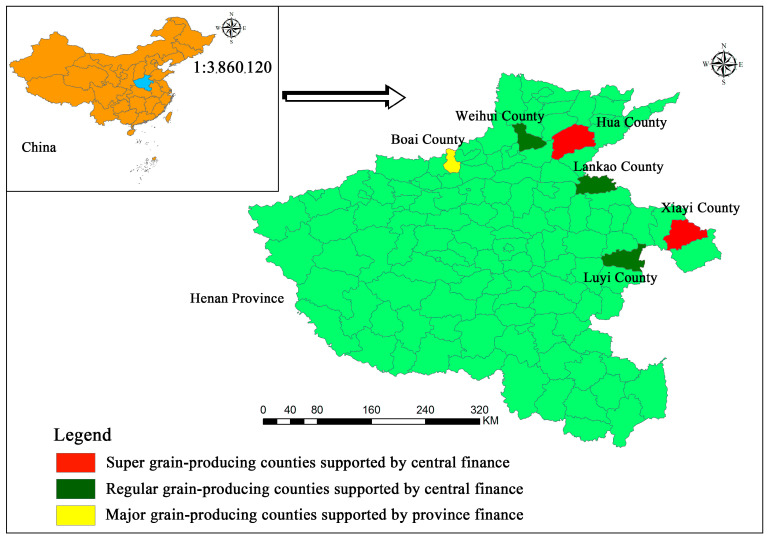
Map of six major grain-producing counties selected in this study.

Henan province is one of the major grain-producing provinces in China. According to the China statistical yearbook-2019, Henan’ total grain output in 2018 was 66.489 million tons, of which the total wheat output was 36.029 million tons, ranking the first in China. The data in this study came from the field survey of 645 farmers in major grain-producing counties in Henan province of China conducted by the research group in April to May 2019. In order to guarantee the rationality of the selected research areas, according to the super grain-producing counties, regular grain-producing counties, and provincial grain-producing counties published by the financial department of Henan province in December 2018 [57]. This study extracted six major grain-producing counties including two super grain-producing counties, three regular grain-producing counties and one provincial grain-producing county. Among them, the super major grain-producing counties were the Huaxian county and Xiayi county; regular major grain-producing counties were Lankao county, Luyi county and Weihui county; provincial major grain-producing county was Boai county. The selected counties can well reflect the situation of agricultural production, ecological protection and others in Henan province. The sample area is shown in Figure 2.

According to the sampling principle stated by Sharafi et al. (2018) [58] and Sharifzadeh et al. (2019) [59], the multi-stage sampling method was adopted in order to ensure the survey quality as follows: Firstly, different sample townships (towns) were selected from each sample county according to the economic conditions and distance, and a total of 12 townships (towns) were selected; Then, different sample villages from each sample township (town) were further selected, and a total of 24 administrative villages were chosen (the sample size of selected villages was determined according to the proportion of the total number of grain farmers in each village); Finally, 25–30 farmers were selected from each sample village according to the number of households and population status (coordinate surveys with each village committee member to ensure that sample farmers were willing to participate in the project).

Considering the cultural level and cognitive ability of the farmers, semi-structured household interview was adopted as the survey mode, and the questionnaires were completed in the form of “question and answer” by the investigators uniformly trained by the research group. A total of 660 questionnaires were issued, and 635 effective questionnaires were eventually collected after deleting questionnaires that were invalid and missing key variables. The effective questionnaires was 96.21%. 

### 3.2. Measurement

The scale consisting of 21 measurement items was developed based on the TPV, the design concept of questionnaires in relevant fields [3,60], the semi-structured household interviews and the actual situation of the PPWs disposal in the research area. Likert 5-point scale was used for each measurement item, ranging from “strongly disagree” to “strongly agree” with values of 1, 2, 3, 4 and 5. In this research, a total of 5 variables were measured, all of which were latent variables and measured by multi-item scales. Table 1 presents all the variables and measurement items.

It is worth noting that this study refined the measurement items of all variables in three steps in order to improve the measurement accuracy of the scale. Firstly, an English version questionnaire was developed and translated into Chinese, and then the measurement items of all variables were slightly modified to adapt to the current research background in China. Secondly, three experts and five graduate students from related research fields were invited to discuss each measurement item several times to ensure the content validity of the questionnaire. After that, based on the feedback of the subjects, some wording of the scale was adjusted in time to make it easier for the sample farmers to read and understand. Finally, before the formal survey, we also conducted a pre-test to verify and modify the measurement items of the questionnaire.

### 3.3. Model Design

**Figure 3:**
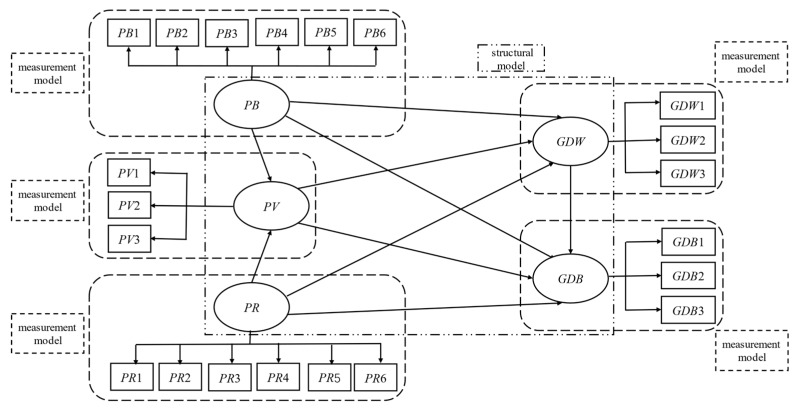
Structural equation model of the constructed theoretical model in this research.

The theoretical model constructed in this research is to explore the causal path and functional relationship among abstract variables in farmers’ PPWs green disposal behavior decision-making, whose essence is a Structural Equation Modeling (SEM) as shown in Figure 3 [32]. In the SEM, the 21 measurement items (*PB*1*-PB*6*, PR*1*-PR*6*, PV*1*-PV*3*, GDW*1*-GDW*3*, GDB*1*-GDB*3) were the observable variables, and the 5 model variables (*PB*, *PR*, *PV*, *GDW*, *GDB*) were considered as the latent variables. The causal path relationship of the 5 latent variables constituted the SEM structural model, and the relationship between latent variables and their corresponding observed variables constituted the SEM measurement model. The regression equations of each model are as follows,

Regression equation of structural model:

(1) $$\eta_{2}=\gamma \xi +\beta \eta_{1}+\zeta$$

Regression equation of the measurement model:

(2) $$X=\lambda_{x}\xi +\delta$$

(3) $$Y=\lambda_{y}\eta +\varepsilon$$

where, η is the endogenous latent variable, ξ is the exogenous latent variable, γ is the estimated parameter, β is the regression coefficient, X is the endogenous variable, namely the independent variable, Y is the exogenous variable, namely the dependent variable, $\lambda_{x}$ is the correlation coefficient matrix between the exogenous latent variable (ξ), $\lambda_{y}$ is the correlation coefficient matrix between the endogenous latent variable (η), δ is the measurement error of X variable, ε is the measurement error of Y variable.

Based on the above considerations, the empirical research was conducted utilizing the SEM and the statistical software of AMOS 24.0 (SPSS, IBM, Armonk, NY, USA) [32,33,49].

## 4. Results

### 4.1. Demographic Characteristics of the Sample Farmers

As shown in Table 2, the proportion of men is significantly higher than that of women, reaching 73.39% in the sample farmers. In terms of age structure, the middle-aged and elderly are the majority, and the farmers over 41 years old accounted for 71.50%, which indicated that current serious situation of rural farming population aging and a large number of rural young adult migrant working. In terms of cultural structure, the education level of the sample farmers was generally low, with 84.88% of them only having a middle school education or below. In addition, the sample families with the labor force below 3 accounted for 68.03%, with arable land less than 0.67 hm^2^ accounting for 69.45%, farming experience more than 20 years accounting for 68.98%, the agricultural income accounted for less than 40% of the annual family income accounting for 66.30%, which to some extent reflected the rural agriculture labor shortage, the obvious degree of part-time employment, and the need to strength the scale agriculture production.

### 4.2. Reliability and Validity Test

This study tested the reliability and validity of the scale. As shown in Table 3, the Cronbach’s alpha values and composite reliability values of all the variables were all significantly higher than the recommended threshold of 0.7 [33], so the reliability of the questionnaire is verified. The convergence validity was tested through the average variance extraction (AVE) of each latent variable and the factor loading of each measurement item [64]. The AVEs values of all the variables (except *PB*) were higher than the recommended threshold value of 0.5, and the standardized factor loading values of all the measurement items are also significantly higher than the recommended threshold value of 0.5 [65]. These results revealed the better single-dimensionality and convergent validity of the questionnaire [66].

In addition, the discriminant validity of each variable was tested. Liu et al. pointed out that the discriminant validity referred to the comparative relationship between the common variance and AVE_S_ value of each variable [64]. As shown in Table 4, the square root of each AVE value was higher than the correlation coefficient of each variable, so the discriminant validity is supported [67]. In view of the above test results, the questionnaire data were stable and reliable, with good convergent validity and discriminant validity.

### 4.3. Model Fitness Test

Considering that there existed reasonable covariant relationships between the variance of variables in the initial theoretical model, a total of 6 covariant relationships including e2 and e3, e8 and e9, e10 and e11, e11 and e12, e10 and e12, e14 and e15 were added, which effectively reduced the chi-square value of the model without going against the theoretical hypothesis [32]. On this basis, the goodness of model fit was calculated as shown in Table 5. A total of 10 indexes including the absolute fitness index, value-added fitness index, simplified fitness index were all in line with the fitness test standard [45], which indicated that the overall fitting degree of the model was good.

### 4.4. Hypothesis Test

Table 6 represented the test results of SEM model hypotheses. The path coefficient between perceived benefits, perceived risks and perceived value of the PPWs green disposal was 0.775 and −0.273, respectively, both of which were significant at *p* < 0.001. This indicated that perceived benefits have a significantly positive impact on perceived value, and perceived risks have a significantly negative impact on perceived value. Accordingly, H1 and H2 were verified. The path coefficient between perceived value, perceived benefits, perceived risks and green disposal willingness of PPWs was 0.250, 0.245, −0.139 and were significant at *p* < 0.001, *p* < 0.001, *p* < 0.01, respectively. This indicated that perceived value has a significantly positive impact, perceived benefits have a significantly positive impact and perceived risks have a significantly negative impact on the green disposal willingness, so H3, H4 and H5 were supported. The path coefficient between perceived value, perceived benefits, perceived risks and the green disposal behaviors of PPWs was 0.358, 0.279 and −0.399 and were significant at *p* < 0.01, *p* < 0.05, *p* < 0.001, respectively. This indicated that perceived value has a significantly positive impact, perceived benefits have a significantly positive impact and perceived risks have a significantly negative impact on the PPWs green disposal behaviors, thus, H6, H7 and H8 were verified. It was worth noting that the path coefficient between the green disposal willingness and behaviors of PPWs was −0.190, which indicated that the green disposal willingness has a negative impact on the behaviors, so H9 was not supported. This also showed that there existed inconsistence between the famers’ PPWs green disposal willingness and behaviors.

Table 7 showed the direct effect, indirect effect and total effect among each variable in the SEM. Firstly, in terms of the perceived value, the impact from perceived benefits (0.633) was the highest, while the impact from perceived risks (−0.279) was the lowest. Secondly, in terms of the green disposal willingness, farmers’ perceived benefits and perceived risks could have important impacts on their PPWs green disposal willingness directly and indirectly, where the total effects of perceived benefits (0.478) were the highest followed by perceived value (0.334) and perceived risks (−0.284). Finally, in terms of the green disposal behaviors, farmers perceived benefits and perceived risks could have significant impacts on the green disposal behaviors of PPWs directly and indirectly, where the directly negative effect (−0.384) of perceived risks was greater than the directly positive effect (0.215) of perceived benefits. Moreover, the negative total effect of perceived risks (−0.362) on the green disposal behaviors through the green disposal willingness was in turn greater than the positive total effect of perceived value (0.299) on the green disposal behaviors through the green disposal willingness and perceived benefits (0.184) on the green disposal behaviors through the green disposal willingness. Notably, the overall effect (−0.116) of the green disposal willingness on the green disposal behaviors was relatively minimal, which indicated that the farmers’ PPWs green disposal willingness to a large extent could not be translated into the actual PPWs green disposal behaviors.

## 5. Discussion

In the context of an increasingly severe pollution of PPWs, this study, based on the TPV, investigated the impacts of farmers’ perceived value on their willingness and behaviors in the PPWs green disposal. In the extant literature, targeted studies, focusing on the farmers’ PPWs green disposal willingness and behaviors, have never been reported. This study found that the TPV was an effective theoretical basis to explain the farmers’ PPWs green disposal willingness and behaviors, which made some theoretical contributions. This provided a new insight to the promotion of grain farmers’ PPWs green disposal willingness and behaviors in Henan province of China, and also a new idea for improving and formulating relevant agricultural pollution prevention policies.

The present research showed that farmers’ perceived value was the result of their comprehensive tradeoff and comparison of perceived benefits and perceived risks of PPWs green disposal. It was further concluded that perceived benefits have more impacts on PPWs green disposal perceived value than perceived risks, which was supported by existing research findings on crop straw and livestock manure [31]. As “rational economic man”, farmers’ behavioral decisions were always based on the prediction of the consequences of behavioral choices (such as the land investment behaviors and the adoption of sustainable farming practices), and they make choices they believe can maximize profits with the minimum risks [43,68]. Dessart et al. [42] pointed out that the financial risks perceived by farmers in agricultural production activities, related to pest control and pesticide use, may be one of the most important obstacles to their adoption actions. While Jin et al. [9] argued that PPWs recycling program should be established through the institutional innovation utilizing the existing economic structure, where all the stakeholders (including farmers) could get a return on the investment. According to the above findings, improving farmers’ perceived benefits of PPWs green disposal and reducing the perceived risks of PPWs green disposal is predicated to improve the perceived value of PPWs green disposal.

The results also suggested that perceived value (0.334) was the most direct factor influencing farmers’ PPWs green disposal willingness. Nonetheless, farmers’ perceived benefits (0.478) were the most important factor influencing their PPWs green disposal willingness (Table 7). The possible explanation was that farmers believed that green disposal of PPWs could increase economic income, reduce environmental pollution and improve health, and this kind of perceived gains could affect farmers’ PPWs green disposal willingness through the positive recognition of the value perception. Research indicated that farmers’ expectations of economic benefits (such as labor saving, high productivity and high returns) are more likely to promote their willingness to engage in environmentally friendly activities [47]. Therefore, in agricultural production activities, improving farmers’ perceived benefits and perceived value could promote the improvement of farmers’ PPWs green disposal willingness, which was consistent with previous research conclusions on the impact of information transfer on farmers’ uptake of innovative crop technologies [69]. Hurley and Mitchell [70] also pointed out that only when farmers understood that the field returns and provides value, can they be motivated to make economic disposal decisions regarding the neonicotinoid seed treatments.

In addition, the results showed that the farmers’ green disposal willingness has a negative impact on the green disposal behaviors of PPWs, which was contrary to our theoretical expectations but interesting to explain. Farmers were worried that PPWs green disposal could not be supported by more policy subsidies, and they would have to invest some extra money, while PPWs green disposal could only generate a little economic income. Therefore, farmers think that it would be better to earn money by going out to work than to spend labor time on PPWs green disposal. Meanwhile, farmers were prone to the inertial discarded behavior due to herd mentality [26], and they realized that PPWs green disposal has significant positive externalities, such as ecological environment protection, safety and health, etc., but they would not dispose PPWs in a green way driven by profit maximization. This was consistent with the existing research results related to diversified agricultural system and farmers’ risk behavior [46,71]. Trujillo-Barrera et al. [47] indicated that the increase in farmers’ risk awareness would not only directly reduce the opportunity to adopt sustainable practices, but also weaken the effect of expected economic returns brought by the adoption of sustainable practices. The empirical results of this study showed that farmers’ perceived benefits showed the greatest total effect on their PPWs green disposal willingness, while perceived risks showed the greatest total effect on their PPWs green disposal behaviors. The findings were consistent with the conclusion of previous studies that “farmers generally have risk aversion psychology when facing to the behavioral choices” [72,73]. To a large extent, this hindered the transformation of farmers’ green disposal willingness into the actual green disposal behaviors, and farmers often showed the “powerless” state in terms of PPWs green disposal behaviors. Therefore, effective and sustainable practices have been adopted to improve farmers’ perceived benefits (especially economic income benefits) and reduce perceived risks (especially cost input risks), which is conducive to the transformation of farmers’ willingness of PPWs green disposal into practical actions.

It should also be pointed out that existing literature pointed out that most farmers usually discarded agricultural waste, such as crop straw, livestock manure and so on, in their fields or around arable land [11,31]. This was similar to the behavioral way the sample farmers disposed of PPWs and its consequences in this study, that is, these inappropriate disposal behaviors seriously threatened the agricultural ecological environment. However, previous studies have never explored the specific behaviors of PPWs “green” disposal, and the impact of perceived value and its influencing factors, namely perceived benefits and perceived risks, on farmers’ PPWs green disposal willingness and behaviors have not been investigated. Marnasidis et al. [60] pointed out that the environmental pollution caused by improper disposal of PPWs became increasingly serious, but the in-depth researches from the micro level, including pesticide bottles were still absent [9]. Therefore, this paper explored farmers’ green disposal willingness and behaviors of PPWs, in terms of the formation mechanism of perceived value, which theoretically made up for the research deficiencies in the related field of farmers’ behaviors. In addition, the conclusion of “the farmers’ inconsistence between PPWs green disposal willingness and behaviors” extended the applicability of the extant behavior theory from a new perspective.

## 6. Conclusions

Exploring the green disposal willingness from the formation mechanism of perceived value is helpful for farmers to dispose PPWs in a green way. In this study, based on the first-hand data of 635 farmers in grain-producing counties in Henan province of China, we introduced the perceived value and its influencing factors, namely perceived benefits and perceived risks, to investigate their influence on the willingness and behaviors of farmers PPWs green disposal. The conclusions were as follows:

(1) The theoretical model of this study based on TPV effectively explained the farmers’ green disposal willingness and behaviors of PPWs. This is because farmers’ green disposal action logic followed the path pattern: perceived value→behavior willingness→behavior performance, where farmers’ perceived value was the result of the tradeoff and comparison between the perceived benefits and perceived risks. Moreover, it was further found that the perceived benefits have a greater impact on the PPWs green disposal perceived value than the perceived risks.

(2) Farmers’ perceived benefits and perceived risks have significantly direct and indirect impacts on their green disposal willingness and behaviors of PPWs, among which the perceived benefits have the greatest positive total effect on farmers’ willingness and the perceived risks have the greatest negative total effect on the behaviors. This indicated that farmers’ perceived risks was the most important factor affecting their PPWs green disposal, and the perceived risks have greater influence than the perceived benefits when farmers make real decisions in the PPWs green disposal.

(3) Inconsistence existed between the farmers’ green disposal willingness and behaviors of PPWs. When faced with the choice of PPWs green disposal, farmers generally have the mentality of risk aversion, which largely hindered the transformation of PPWs green disposal willingness into actual green disposal behaviors. Furthermore, driven by the profit maximization, farmers were prone to conservative disposal behaviors and even showed the “powerless” state where they had willingness but no actual action.

### 6.1. Policy Implications

This study provided some important guidelines on PPWs green disposal policy. Firstly, given the importance of perceived value on the PPWs green disposal willingness and behaviors of farmers, local governments should strengthen the publicity and education of the PPWs green disposal, especially clarifying the relationship between the PPWs green disposal and ecological environmental protection along with safety and health, which could improve the farmers’ perceived value level of PPWs green disposal. Secondly, because the perceived benefits and perceived risks have significant effects on the farmers willingness and behaviors of PPWs green disposal, the governments should increase the intensity of policy incentives, such as the implementation of agricultural subsidies, environmental awards or other preferential policies for green disposal behaviors. This could ensure the investment needed by farmers to implement PPWs green disposal, thus reducing the cost risk of green disposal. Finally, some inconsistencies existed between farmers’ green disposal willingness and behaviors. Authorities should take some powerful measures to promote the actual transformation of green disposal willingness to green disposal behaviors, such as encouraging agricultural materials supplier and waste recycling enterprises to actively participate in, building up the transparent and efficient platform for the PPWs green disposal and the reverse recovery [9], promoting the PPWs marketization trade. Meanwhile, the PPWs green disposal could be promoted by taking the local large growers as the entry point, and consciously driven by the informal experience exchange among villagers.

### 6.2. Future Research

Despite the in-depth research, there are some issues that deserve further exploration in the future. Firstly, this paper only investigated the impact of farmers’ perceived value on their green disposal willingness and behaviors of PPWs, and future studies should explore more possible influencing mechanisms to improve the research framework. The ability and opportunity [74] may be two important variables for green disposal willingness and perhaps hold a stronger explanation for the green disposal behaviors. Future studies should consider the two variables as the antecedent variable to analyze the underlying reason for the inconsistence between the PPWs green disposal willingness and behaviors. Secondly, this study did not consider the influence of moderator variables, such as policy regulation on the PPWs green disposal willingness and behaviors of farmers. It was pointed out that institutional situations can often moderate the effects of individual behavioral willingness on behavioral decision-making [31]. Therefore, multi-group SEM can be adopted in future research, and policy regulation can be introduced as the moderator variable to analyze the moderating effect of policy regulation on farmers’ green disposal willingness and behaviors of PPWs. Thirdly, the research conclusions were based on the survey data of 635 grain farmers in six major grain-producing counties in Henan province of China, and whether the research conclusions can be extended to farmers in different crops and regions remains to be verified. Future research should expand the survey scope for different types of farmers and supplement more survey samples with different regional attributes.

## Figures and Tables

**Table 1:** Variables and measurement items.

Variables	Measurement Items	Scale Sources
Perceived Benefits(*PB*)	*PB*1.I think selling pesticide packaging waste to recycling buyers can generate some economic income	Han [31] and Wang et al. [40]
	*PB*2.I think the green disposal of pesticide packaging waste can reduce water, soil and other pollution	
	*PB*3.I think the green disposal of pesticide packaging waste is beneficial to the safety of agricultural products	
	*PB*4.I think the green disposal of pesticide packaging waste can improve living environment and benefit physical and mental health	
	*PB*5.I know some of the techniques for green disposal of pesticide packaging waste and have introduced them to my neighbors	
	*PB*6.I think green disposal of pesticide packaging waste saves resources and wins social recognition	
Perceived Risks(*PR*)	*PR*1.Using advanced technology to treat pesticide packaging waste in a green way requires more investment	Ren et al. [43], Menapace et al. [61] and Fahad et al. [62]
	*PR*2.I have to spend time learning about the knowledge and techniques of green disposal of pesticide packaging waste	
	*PR*3.It is more profitable to work in the city than to spend time and energy on the green disposal of pesticide packaging waste	
	*PR*4.I am concerned that there is no policy support or technology for green disposal of pesticide packaging waste	
	*PR*5.I am worried that the green disposal of pesticide packaging waste will be opposed by my neighbors and family	
	*PR*6.I am worried that it will be difficult for the restoration after the destruction of agriculture ecology environment.	
Perceived Value(*PV*)	*PV*1.I have a certainly attitude towards the green disposal of pesticide packaging waste	Sweeney and Soutar [37] and Shannon [54]
	*PV*2.I think green disposal of pesticide packaging waste has positive significance	
	*PV*3.I think green disposal of pesticide packaging waste can bring benefits	
Green Disposal Willingness(*GDW*)	*GDW*1.I am willing to invest labor in green disposal of pesticide packaging waste	De Leeuw et al. [45] and Senger et al. [30]
	*GDW*2.I am willing to invest time in green disposal of pesticide packaging waste	
	*GDW*3.I am willing to invest money in green disposal of pesticide packaging waste	
Green Disposal Behaviors(*GDB*)	*GDB*1.I have put in some labor to green dispose of pesticide packaging waste	Pratiwi et al. [41] and Bagheri et al. [63]
	*GDB*2.I have put in some time to green dispose of pesticide packaging waste	
	*GDB*3.I have put in some money to green dispose of pesticide packaging waste	

**Table 2:** Statistical characteristics of the sample farmers.

Characteristics	Categories	*N*	%	Characteristics	Categories	*N*	%
Gender	Male	466	73.39	Arable area	<0.20 hm^2^	80	12.60
	Female	169	26.61		0.20–0.33 hm^2^	135	21.26
Age (years)	<30	44	6.93		0.33–0.67 hm^2^	226	35.59
	30 to 40	137	21.57		0.67–1.33 hm^2^	152	23.94
	40 to 50	151	23.78		≥1.33 hm^2^	42	6.61
	50 to 60	186	29.29	Farming experience (years)	<10	95	14.96
	≥60	117	18.43		10–20	102	16.06
Education Attainment	Illiteracy	96	15.12		20–30	225	35.43
	Primary school	194	30.55		30–40	164	25.83
	Secondary school	249	39.21		≥40	49	7.72
	High school	54	8.51	Ratio of farm income to total income	0–20%	207	32.60
	College and above	42	6.61		20–40%	214	33.70
Household labor force (persons)	≤1	60	9.45		40–60%	151	23.78
	2–3	372	58.58		60–80%	52	8.19
	≥4	203	31.97		80–100%	11	1.73

**Table 3:** Results of the reliability and validity test (*N* = 635).

Variables	Measurement Items	Mean	Standardized Factor Loading	Cronbach’s Alpha	Composite Reliability	AVE
Perceived Benefits (*PB*)	*PB*1	3.414	0.633 ***	0.838	0.842	0.473
	*PB*2	3.816	0.698 ***			
	*PB*3	3.443	0.549 ***			
	*PB*4	3.980	0.729 ***			
	*PB*5	3.885	0.742 ***			
	*PB*6	3.613	0.753 ***			
Perceived Risks (*PR*)	*PR*1	4.054	0.689 ***	0.859	0.867	0.522
	*PR*2	4.041	0.685 ***			
	*PR*3	4.106	0.681 ***			
	*PR*4	3.373	0.825 ***			
	*PR*5	3.321	0.793 ***			
	*PR*6	3.117	0.645 ***			
Perceived Value (*PV*)	*PV*1	3.731	0.761 ***	0.849	0.850	0.653
	*PV*2	3.531	0.832 ***			
	*PV*3	3.616	0.830 ***			
Green Disposal Willingness (*GDW*)	*GDW*1	4.180	0.786 ***	0.819	0.850	0.656
	*GDW*2	4.006	0.920 ***			
	*GDW*3	3.639	0.710 ***			
Green Disposal Behaviors (*GDB*)	*GDB*1	1.961	0.899 ***	0.872	0.881	0.714
	*GDB*2	1.887	0.688 ***			
	*GDB*3	1.940	0.928 ***			

Note: Significant at *** *p* < 0.001.

**Table 4:** Results of discriminant validity (*N* = 635).

Variables	Means	SD	*PB*	*PR*	*PV*	*GDW*	*GDB*
Perceived Benefits	3.692	0.841	**0.688**				
Perceived Risks	3.669	0.900	0.619	**0.722**			
Perceived Value	3.626	0.972	0.680 **	−0.626 **	**0.808**		
Green Disposal Willingness	3.942	0.900	0.669 **	−0.571 **	0.676 **	**0.810**	
Green Disposal Behaviors	1.929	0.984	0.118 *	−0.117 **	0.132 **	−0.115 *	**0.845**

Note: The square roots of AVE_S_ are the bold elements; Significant at ** *p* < 0.01, * *p* < 0.05.

**Table 5:** Results of model fitness test (*N* = 635).

Goodness-of-Fit Index	Statistical Test Index	Model Estimate	Judgement Standard	Test Result
Absolute fitness index	X^2^/DF	3.915	<5	Accepted
	GFI	0.907	>0.9	Accepted
	RMSEA	0.068	<0.08	Accepted
Value-added fitness index	NFI	0.913	>0.9	Accepted
	IFI	0.934	>0.9	Accepted
	TLI	0.920	>0.9	Accepted
	CFI	0.934	>0.9	Accepted
Simplified fitness index	PGFI	0.680	>0.5	Accepted
	PNFI	0.752	>0.5	Accepted
	CAIC	1109.67 < 1721.787 1109.67 < 7981.686	the theoretical model is smaller than both the saturation model and the independent model	Accepted

Note: X^2^/DF, GFI, RMSEA, NFI, IFI, TLI, CFI, PGFI, PNFI and CAIC mean ratio of chi-square to degrees of freedom, goodness-of-fit degree index, root mean square error of approximation, normed fit index, incremental fit index, non-normed fit index, comparative fit index, parsimony goodness-of-fit index, parsimony-adjusted NFI, consistent Akaike information criterion, respectively.

**Table 6:** Results of SEM model hypothesis test (*N* = 635).

Hypothesis				Unstandardized Coefficients	*t*-Values	Standardized Coefficients	Results
H1	Perceived Value (PV)	<---	Perceived Benefits	0.775	10.453 ***	0.633	supported
H2		<---	Perceived Risks	−0.273	−5.195 ***	−0.279	supported
H3	Green Disposal Willingness (GDW)	<---	Perceived Value	0.250	3.806 ***	0.334	supported
H4		<---	Perceived Benefits	0.245	3.299 ***	0.267	supported
H5		<---	Perceived Risks	−0.139	−3.172 **	−0.191	supported
H6	Green Disposal Behaviors (GDB)	<---	Perceived Value	0.358	2.958 **	0.338	supported
H7		<---	Perceived Benefits	0.279	2.053 *	0.215	supported
H8		<---	Perceived Risks	−0.399	−4.773 ***	−0.384	supported
H9		<---	Green Disposal Willingness	−0.190	−1.783	−0.116	Not supported

Note: ***, ** and * mean significant at *p* < 0.001, *p* < 0.01, *p* < 0.05, respectively.

**Table 7:** Standardized direct effect, indirect effect and total effect between variables (*N* = 635).

Path Relationship			Direct Effect	Indirect Effect	Total Effect
Perceived Value (PV)	<---	Perceived Benefits	0.633	—	0.633
	<---	Perceived Risks	−0.279	—	−0.279
Green Disposal Willingness (GDW)	<---	Perceived Value	0.334		0.334
	<---	Perceived Benefits	0.267	0.211	0.478
	<---	Perceived Risks	−0.191	−0.093	−0.284
Green Disposal Behaviors (GDB)	<---	Perceived Value	0.338	−0.039	0.299
	<---	Perceived Benefits	0.215	−0.031	0.184
	<---	Perceived Risks	−0.384	0.022	−0.362
	<---	Green Disposal Willingness	−0.116	—	−0.116

Note: Indirect effect of perceived benefits→green disposal willingness = 0.633 × 0.334 ≈ 0.211; Indirect effect of perceived risks→green disposal willingness = (−0.279) × 0.334 ≈ −0.093; Indirect effect of perceived benefits→green disposal behaviors = 0.267 × (−0.116) ≈ −0.031; Indirect effect of perceived risks→green disposal behaviors = (−0.191) × (−0.116) ≈ 0.022; Indirect effect of perceived value→green disposal behaviors = 0.334 × (−0.116) ≈ −0.039; Total effect = direct effect + indirect effect.
